# Interobserver agreement on line-field confocal optical coherence tomography image markers in keratinocyte carcinomas and precursor lesions

**DOI:** 10.1007/s00403-024-03344-y

**Published:** 2024-09-06

**Authors:** Kevin Jacobsen, Vinzent Kevin Ortner, Emily Wenande, Gabriella Fredman, Gavrielle R. Untracht, Tom Wolswijk, Emmy Cruts, Klara Mosterd, Kari Nielsen, Peter Alshede Philipsen, Stine Regin Wiegell, Merete Haedersdal

**Affiliations:** 1 Department of Dermatology, Copenhagen University Hospital – Bispebjerg and Frederiksberg, Copenhagen, Denmark; 2 Department of Dermatology, Maastricht University Medical Center+, Maastricht, The Netherlands; 3 GROW Research Institute for Oncology and Reproduction, Maastricht University, Maastricht, The Netherlands; 4 Department of Dermatology, Clinical Sciences Lund, Lund University and Skaane University Hospital, Lund, Sweden; 5 Department of Clinical Medicine, University of Copenhagen, Copenhagen, Denmark; 6 Department of Health Technology, Technical University of Denmark, Kongens Lyngby, Denmark

**Keywords:** Line-field confocal optical coherence tomography, Basal cell carcinoma, Squamous cell carcinoma, Carcinoma in situ, Actinic keratosis, Keratinocyte carcinoma, Interobserver agreement

## Abstract

Line-field confocal optical coherence tomography (LC-OCT) is a new technology for skin cancer diagnostics. However, the interobserver agreement (IOA) of known image markers of keratinocyte carcinomas (KC), including basal cell carcinoma (BCC) and squamous cell carcinoma (SCC), as well as precursors, SCC in situ (CIS) and actinic keratosis (AK), remains unexplored. This study determined IOA on the presence or absence of 10 key LC-OCT image markers of KC and precursors, among evaluators new to LC-OCT with different levels of dermatologic imaging experience. Secondly, the frequency and association between reported image markers and lesion types, was determined. Six evaluators blinded to histopathologic diagnoses, assessed 75 LC-OCT images of KC (21 SCC; 21 BCC), CIS (12), and AK (21). For each image, evaluators independently reported the presence or absence of 10 predefined key image markers of KCs and precursors described in an LC-OCT literature review. Evaluators were stratified by experience-level as experienced (3) or novices (3) based on previous OCT and reflectance confocal microscopy usage. IOA was tested for all groups, using Conger’s kappa coefficient (κ). The frequency of reported image marker and their association with lesion-types, were calculated as proportions and odds ratios (OR), respectively. Overall IOA was highest for the image markers *lobules* (κ = 0.68, 95% confidence interval (CI) 0.57;0.78) and *clefting* (κ = 0.63, CI 0.52;0.74), typically seen in BCC (94%;OR 143.2 and 158.7, respectively, *p* < 0.001), followed by *severe dysplasia* (κ = 0.42, CI 0.31;0.53), observed primarily in CIS (79%;OR 7.1, *p* < 0.001). The remaining seven image-markers had lower IOA (κ = 0.06–0.32) and were more evenly observed across lesion types. The lowest IOA was noted for a *well-defined* (κ = 0.07, CI 0;0.15) and *interrupted dermal-epidermal junction* (DEJ) (κ = 0.06, CI -0.002;0.13). IOA was higher for all image markers among experienced evaluators versus novices. This study shows varying IOA for 10 key image markers of KC and precursors in LC-OCT images among evaluators new to the technology. IOA was highest for the assessments of *lobules*,* clefting*, and *severe dysplasia* while lowest for the assessment of the DEJ integrity.

**Supplementary Information:**

The online version contains supplementary material available at 10.1007/s00403-024-03344-y.

## Introduction

### Skin cancer burden and the need for non-invasive imaging tools

Skin cancer is the most frequent cancer worldwide [1] and keratinocyte carcinomas (KC), including basal cell carcinoma (BCC) and squamous cell carcinoma (SCC), are the most common subtypes [2, 3]. The past decades have seen a marked increase in rates of KC and its precursors, SCC in situ (CIS) and actinic keratosis (AK) [4]. Consequently, new, less time- and resource-consuming non-invasive methods are needed for skin cancer detection. Such advances are expected to enhance the patient experience, and mitigate healthcare costs, particularly in high-risk populations that often require intensified KC management, including organ transplant recipients (OTR) [5]. Non-invasive imaging techniques, such as optical coherence tomography (OCT) and reflectance confocal microscopy (RCM), are increasingly being implemented in the field of dermato-oncology, due to their ability to visualize skin layers below the skin surface in real-time [6–9]. These techniques are reported to improve the diagnostic accuracy of KC lesion evaluation, especially when used in combination with dermoscopy [7, 10]. Moreover, imaging enables monitoring of KC treatment outcomes [11, 12], and enhances preoperative surgical planning [6, 13], underscoring its broad potential in skin cancer management.

### Line-field confocal optical coherence tomography

Line-field confocal optical coherence tomography (LC-OCT) was introduced in 2018 as a non-invasive imaging technique based on the principles of OCT and RCM [14, 15]. As such, LC-OCT combines high image resolution, depth penetration, and imaging speed to generate real-time three-dimensional (3D) multi-planar imaging (*cross-section* and *en-face*) of the epidermis and superficial dermis at the cellular level. The modality is further enhanced by a dermoscopy-like camera, enabling simultaneous visualization of the skin surface to target the precise region of interest [16]. Since its introduction, numerous studies have explored the use of LC-OCT to characterize KC and precursor lesions in vivo [17–27].

### LC-OCT image markers and clinical implementation

Implementing LC-OCT into the clinic, includes identification of recognizable image markers within lesions, understanding the level of interobserver agreement (IOA) among individuals reporting on these markers, and understanding their utility for diagnosis. In a recent review, we compiled a comprehensive glossary of LC-OCT markers for KCs and precursors, identifying 10 key image markers for KC, CIS, and AK lesions that were integrated with conventional histopathology [28]. The IOA in which these predefined LC-OCT markers are reported by different evaluators without prior LC-OCT experience remains to be tested.

### Objectives

The primary objective of this study was to determine IOA on the presence or absence of 10 key LC-OCT image markers of BCC, SCC, CIS, and AK among evaluators new to LC-OCT with different experience-levels in dermatologic imaging. For a more comprehensive understanding of factors that may contribute to varying IOAs, the study additionally assessed evaluators’ confidence and opinion on whether image-artefacts impacted individual image marker evaluations. A secondary objective was to determine the frequency of image markers reported by evaluators for each lesion-type and test the association between image marker and lesion-type.

## Methods

### Patients, lesion-types, and LC-OCT images

For this study, LC-OCT images of various BCC, SCC, CIS, and AK lesions were required. Included patients were identified and corresponding images registered in the dedicated OTR clinic at Dermatology Department, Bispebjerg Hospital, Copenhagen, Denmark, between September 2022 and June 2023. Images were collected by a single physician with two years of LC-OCT experience (K.J.) at the time of study initiation. All patients signed informed consent and preapproval for the collection of LC-OCT images was obtained by local ethical committee (H-21038387). The study was conducted in accordance with the Declaration of Helsinki.

### LC-OCT image acquisition

Imaging was performed using a commercially available, hand-held LC-OCT scanner (deepLive™, DAMAE Medical, Paris, France, 800 nm, axial and lateral resolution 1.3 μm; field of view 1.2 mm x 0.5 mm x 0.5 mm). Data was prepared as high-resolution videos using Open Broadcaster Software, version 30 (OBS^®^, Corunna, USA) enabling presentation of 3D image-cases to evaluators on a computer screen i.e., front-to-back in the *cross-section*-view and top-to-bottom in the *en-face-*view (Figure S1).

### Key image markers

**Fig. 1:**
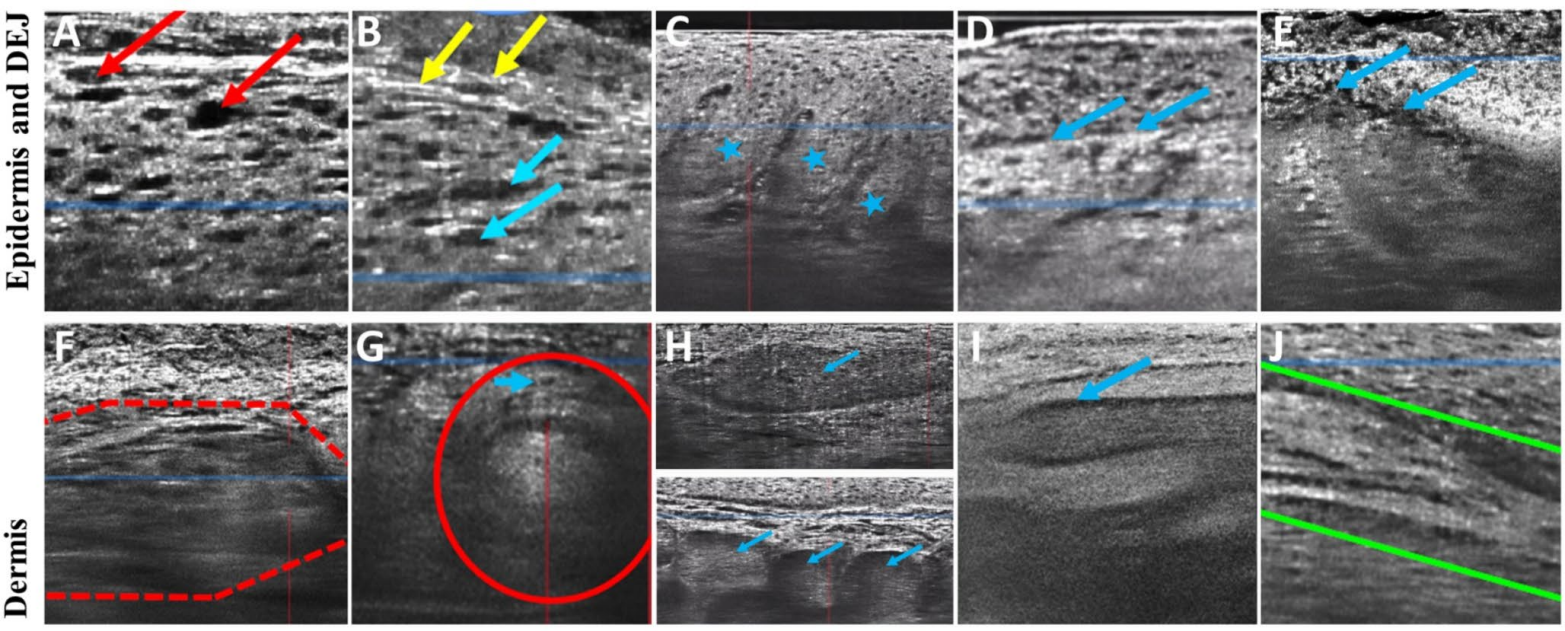
Line-field confocal optical coherence tomography images of 10 key markers and their definition. Illustration and description of 10 predefined and previously reported key image markers evaluated in the study [28]. Epidermal markers A-C and H (top image). Dermal-epidermal junction markers D-E. Dermal markers F-J as well as H (bottom image). Images are resized for illustrative purposes. **A.** Severe dysplasia (i.e., full-thickness epidermal dysplasia), with large, atypical cells (red arrows) in the stratum granulosum layer. **B.** Mild-moderate dysplasia, with large, atypical cells in the stratum spinosum and basal layer (blue arrows) but not in stratum granulosum layer (yellow arrows). **C.** Tumor budding, epidermal protrusion into the dermis (blue stars) composed of disarranged atypical cells with an intact dermal-epidermal junction. **D.** Well-defined dermal-epidermal junction, a clear line separating the epidermis from the dermis (blue arrows). **E.** Interrupted dermal-epidermal junction, the line separating the epidermis is blurry/not visible (blue arrows). **F.** Broad strands, dermal compartment (red dashed line) presenting with a core of disarranged atypical cells. **G.** Keratin pearl, circular bright structure in the dermis with no connection to the epidermis (red circle), surrounded by a layer of atypical cells (blue arrow). **H.** Lobule, hemispheric or circular structures (blue arrows) with an inner millefeuille pattern that is either connected to the epidermis (top) or unconnected to the epidermis (bottom). **I.** Clefting, dark space immediately surrounding the lobule (blue arrow). **J.** Collagen alterations, bright dense structure in the dermis (within green lines)

Ten predefined key LC-OCT image markers identified in a previously published review and correlated to diagnostically important histopathological markers for KC and precursor lesions [28], were evaluated in the study. The markers included epidermal markers (*severe dysplasia (i.e.*,* full-thickness epidermal dysplasia)*,* mild-moderate dysplasia*,* and tumor budding)*, dermal-epidermal markers *(well-defined dermal-epidermal junction (DEJ) and interrupted DEJ)*, and dermal markers *(broad strands*,* keratin pearls*,* clefting*, and *collagen alterations)* and *lobules* located in the epidermis or dermis **(**Fig. 1**).**

### Evaluators, experience and training

Six evaluators, all new to LC-OCT but with varying experience in conventional OCT and/or RCM skin cancer imaging, participated in the study. Evaluators originated from three university hospitals: Maastricht University Medical Center + in the Netherlands, Skaane University Hospital in Sweden, and The Bispebjerg Hospital (BBH) in Denmark. Three evaluators who had completed an e-learning on OCT with continuous monitoring of the ability to differentiate between BCC and non-BCC by cumulative sum analysis [29] were categorized as ‘experienced’ imaging device users; the remaining three were categorized as ‘novices’ (Table S1). To ensure that evaluators were familiar with all 10 image markers, a pictorial overview with brief descriptions for each marker, as illustrated in Fig. 1, was sent to each evaluator a week prior to assessments. On the day of the evaluation, an approximately 2 hour in-person session was held to clarify study procedures and review specific image features, examples of healthy skin and image artifacts (e.g., shadows caused by hyperkeratosis). When referring to the extent of disease, multiple grades could not co-exist within the same image and only one grade was given (i.e., ‘*severe*’ vs. ‘*mild-moderate*’ *dysplasia*, or ‘*well-defined* vs. *interrupted DEJ*’); rather, the highest disease level was reported.

### Image evaluation

Image-cases were presented by a moderator (K.J.). Evaluators assessed each image independently without the possibility of discussion. Evaluators were blinded to the histopathology diagnosis and clinical photos related to the specific lesion. Assessments were conducted in one live meeting (35 cases), and three virtual meetings (40 cases). For each case, both images of the lesion and healthy skin of the same patient were shown. First, a single *cross-section* image of the healthy skin was shown, whereafter the 3D image of the lesion was displayed. For each case and image marker, evaluators responded to the following three questions: (1) *‘is the marker present?’ (yes/no)*, (2) *‘are you confident in your decision?’ (yes/no)*, (3) *‘did artefacts impact your decision?’ (yes/no).* Additional written comments other than yes or no (such as ‘not applicable’) were allowed, if evaluators were unable to make a decision about the presence or absence of an image marker. In the final dataset, missing data was omitted and evaluator responses other than *yes/no* were recoded.

### Outcome measures

The primary outcome measure was IOA in the assessment of 10 LC-OCT markers (Fig. 1), expressed as the Conger’s kappa (κ) coefficient (a generalization of Cohen’s kappa for multiple evaluators) [30]. Reported for all evaluators (*n* = 6) and stratified by experience level (*n* = 3), κ values were interpreted as poor (≤ 0), slight (0.01–0.2), fair (0.21–0.4), moderate (0.41–0.6), substantial (0.61–0.8) or almost perfect (> 0.81) as previously described in the literature [31]. To identify κ paradoxes where high agreement but low κ values coexist [32, 33], other complementary agreement measures were also calculated, including overall agreement (P_o_), agreement on the presence (P_pos_) or absence (P_neg_) of a marker, and reported prevalence on marker presence (f_1_) or absence (f_2_) (described in detail in Table S2).

Evaluators’ responses on confidence levels (i.e., observer’ confidence*)* and influence of image-artefacts (i.e., resistance to image-artefacts) for each marker, were expressed as proportions of *yes/no* responses, and the responses of experienced (*n* = 3) and novice users (*n* = 3) were compared.

Finally, the frequency of reported image markers for each lesion-type for all evaluators (*n* = 6) were expressed as proportions, and the association between the presence of independent image markers and lesion-type were expressed as odds ratios (OR) with 95% confidence intervals (CI).

### Sample size calculation, statistical analyses, and data quality assurance

A sample size calculation for the κ statistics for two evaluators was performed using a validated online tool (https://wnarifin.github.io/ssc/sskappa.html). The minimal accepted κ value was prespecified at 0.4, and the expected κ value to 0.8, with a power of 0.80 and level of significance of 0.05 [34–36]. To estimate the anticipated prevalence rate of each image marker, an experienced LC-OCT user (K.J.), assessed 156 images of KC, CIS, and AK lesion types from a LC-OCT imaging database at BBH. Although the ideal prevalence rate is 50% for a κ statistics sample size calculation [32], the prevalence rate in that dataset was found to be 4-69%. We chose 15% as the prevalence rate for the sample size calculation, resulting in a recommended sample size of 75 images. Included images (*n* = 75) aimed to ensure a prevalence rate as close as possible to 50% for all image markers, as well as acceptable image quality and a balanced distribution of available lesion-types: SCC (8 cases, 21 images), BCC (7 cases, including 6 images of superficial BCC and 15 images of nodular BCC), CIS (4 cases, 12 images) and AK (7 cases, including 6 images of AK clinical grade I, 9 images of AK clinical grade II, 6 images of AK clinical grade III). Six evaluators were later included in the study, exceeding the recommended minimum of two evaluators.

κ with 95% CIs were calculated using ‘simpleagree’ (https://svanbelle.shinyapps.io/simpleagree/) and was chosen over Fleiss’ kappa since the same set of evaluators evaluated all images [37]. Table S2 shows how P_o_, P_pos_, P_neg_, f_1_, and f_2_ were calculated. The proportion of observer’ confidence and resistance to image-artefacts responses between experienced and novice groups for each image marker were compared using the McNemar’s exact test. We used a binary logistic regression model to test whether any of the image markers were associated with specific lesion-types, estimating OR with 95% CI. The model consisted of one binary dependent variable (‘image marker’) and two categorical independent variables (‘single lesion-type’ compared to all other lesion-types, and ‘evaluators’). The dataset for the binary logistic regression analysis included the responses on image markers from all six evaluators for each assessment, coded as 1 if the image marker was present and 0 if absent. Evaluators were included as an independent variable to adjust for differences in their responses. We performed the regression for each of the ten image markers for each combination of the four specific lesion-types. The two latter statistical analyses were performed using SPSS, version 29 (IBM Corp, Chicago, USA). *P* values < 0.05 were considered statistically significant.

## Results

### Overall interobserver agreement

**Fig. 2:**
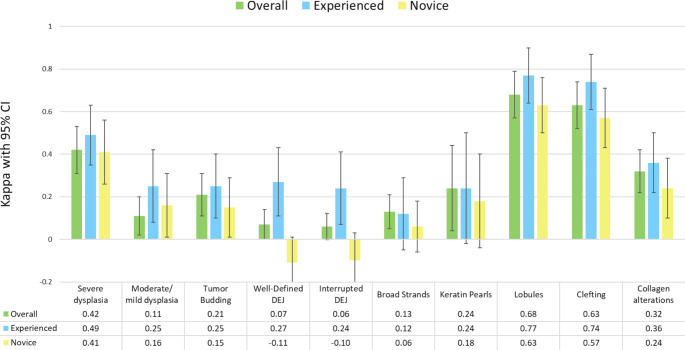
Interobserver agreement as measured by Conger’s kappa of 10 key LC-OCT image markers of keratinocytic lesions and precursors categorized by level of evaluator experience. Bars with 95% confidence intervals (CI) show Conger’s kappa values for all six evaluators with conventional OCT and RCM background experience (green), of which three were categorized as experienced (blue) and three were categorized as novices in non-invasive imaging (yellow)

κ values among the six evaluators in the assessment of 10 different image markers ranged from slight to substantial (0.06–0.68, Fig. 2 and Table S2). The highest κ values were achieved for *lobules* (0.68, 95% CI 0.57;0.78) and *clefting* (0.63, CI 0.52;0.74), followed by *severe dysplasia* (0.42, CI 0.31;0.53). In contrast, the lowest κ values were observed for a *well-defined DEJ* (0.07, CI 0;0.15) and an *interrupted DEJ* (0.06, CI -0.002;0.13).

### Interobserver agreement by experience-level

For all image markers, κ values were higher for experienced evaluators than for novices, most evident for markers related to DEJ integrity (Fig. 2 and Table S2). For both experience groups, the highest κ values were found for *lobules* (experienced 0.77, CI 0.64;0.90 vs. novices 0.63, CI 0.50;0.77), *clefting* (experienced 0.74, CI 0.61;0.87 vs. novices 0.57, CI 0.43;0.71), and *severe dysplasia* (experienced 0.49, CI 0.35;0.64 vs. novices 0.41, CI 0.26;0.55). κ values for a *well-defined DEJ and interrupted DEJ* were particularly low among novices (-0.11, CI -0.23;0.004 and κ = -0.10, CI -0.23;0.02, respectively).

### Observer confidence and resistance to artefacts stratified by experience

**Fig. 3:**
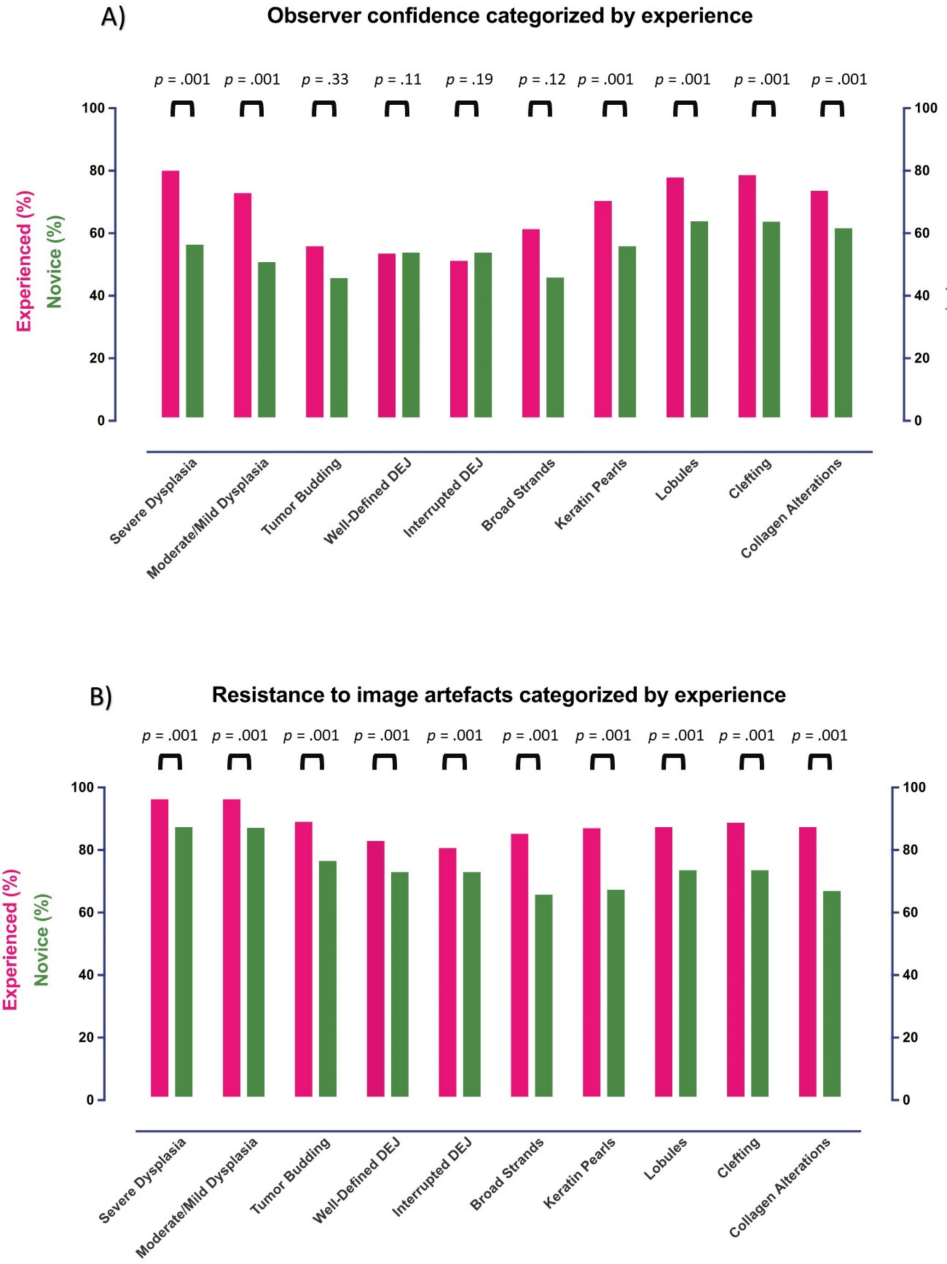
Reported observer confidence and resistance to artefacts for 10 key LC-OCT image markers in images of basal cell carcinoma, squamous cell carcinoma, squamous cell carcinoma in situ/carcinoma in situ, and actinic keratosis. (**A**) Observer confidence (i.e., the proportion of ‘*yes confident*’ responses) for conventional optical coherence tomography and reflectance confocal microscopy experienced (*n* = 3) and novice (*n* = 3) evaluators, respectively. (**B**) Resistance to image-artefacts (i.e., the proportion of ‘*no*,* image artefacts did not impact decision*’ responses) for conventional optical coherence tomography and reflectance confocal microscopy experienced (*n* = 3) and novice (*n* = 3) evaluators, respectively

Experienced evaluators were more confident compared to novices when evaluating six out of 10 image markers (*p* < 0.001), while no difference was observed for the remainder: *tumor budding* (*p* = 0.33), *well-defined DEJ* (*p* = 0.11), *interrupted DEJ* (*p =* 0.19) and *broad strands* (*p* = 0.12) (Fig. 3A**)**. Consistent with IOA findings, experienced evaluators confidence was highest for *severe-dysplasia* (81%), *clefting* (80%), and *lobules* (79%) and lowest for *an interrupted DEJ* (52%) and *well-defined DEJ* (55%).

For all image markers, experienced evaluators were less influenced by artefacts compared to novices (*p* < 0.001) as illustrated in Fig. 3B. Notably, both groups’ assessments of the epidermal markers, *severe dysplasia*,* mild-moderate dysplasia*,* and tumor budding* were reportedly less impacted by artefacts, in contrast to assessments of the dermal markers, *broad strands*,* keratin pearls*,* clefting*, and *collagen alterations*, and the epidermal/dermal marker *lobules*. The image markers most often reported to be influenced by artefacts were an *interrupted DEJ* for the experienced group (resistance to image artefacts, 81.7%) and *broad strands* for the novice group (66.8%).

### Reported image markers by lesion-type

**Fig. 4:**
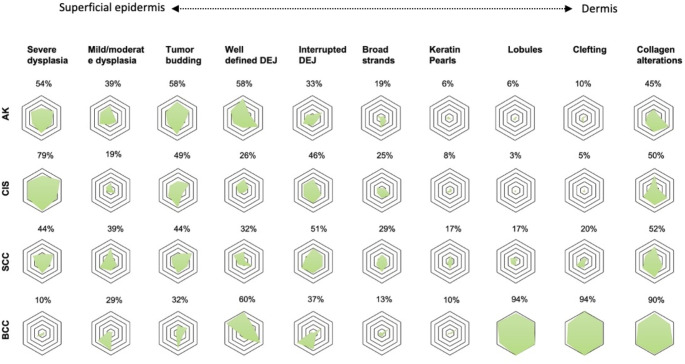
Reported image markers in line-field confocal optical coherence tomography images of keratinocyte carcinomas and precursor lesions. For each image marker, a radar chart is plotted for basal cell carcinoma (BCC), squamous cell carcinoma (SCC), squamous cell carcinoma in situ/carcinoma in situ (CIS), and actinic keratosis (AK). Green shaded areas within radar charts represents the number independent evaluators reported a given image marker in images of BCC, SCC, CIS, and AK, with each of the six corners representing a single evaluator (*n* = 6). The outermost ring represents 100% with 5 lines extending inwards in increments of 20%. Percentages are the mean response for all six evaluators

Certain markers appeared to have greater lesion-specificity than others (Fig. 4**).** The image markers, *lobules and clefting*, were frequently observed in BCCs (94%, 94%, respectively) and less in images of SCC (17%, 20%), CIS (3%, 5%), and AK (6%, 10%). Moreover, the image marker, *severe dysplasia*, was frequently reported in CIS (79%) and more rarely in BCC (10%), SCC (44%), and AK (54%). The remaining image markers were more evenly distributed across all lesion types. For example, an *interrupted DEJ* were reported with similar frequencies for BCC (37%), SCC (51%), CIS (46%), and AK (33%).

**Fig. 5:**
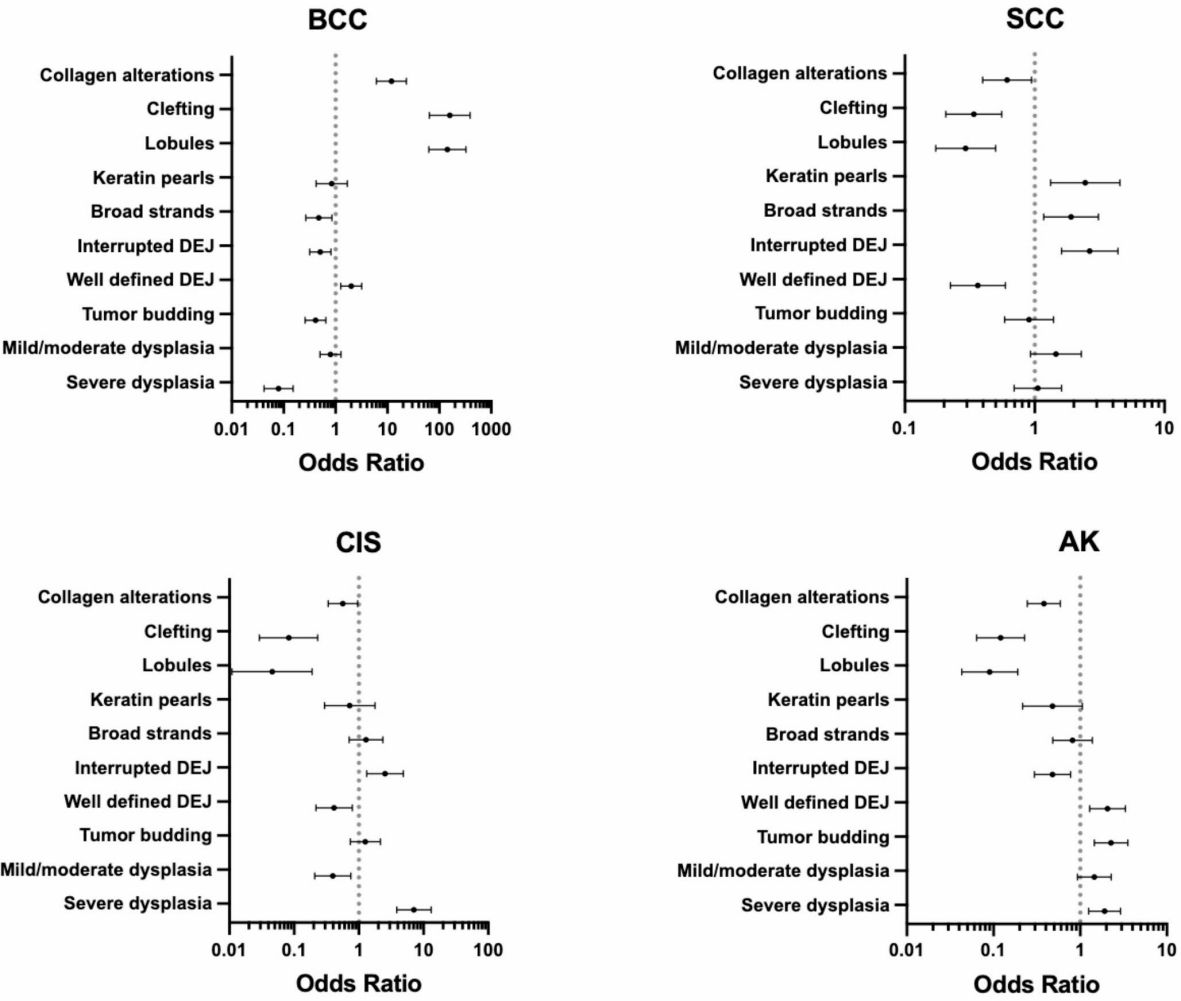
Odds ratios for 10 key image markers reported by evaluators in line-field confocal optical coherence tomography images of basal cell carcinoma (BCC), squamous cell carcinoma (SCC), squamous cell carcinoma in situ/carcinoma in situ (CIS), and actinic keratosis (AK). The association of reported image markers with lesion types are presented as odds ratios (plotted as black dots on individual graphs) with 95% confidence intervals on a log-scale. The independent variable is a single lesion type compared to other lesion-types, adjusted for differences in evaluators’ responses. Odds ratios where the 95% confidence intervals do not intersect the line (OR = 1) indicate a statistically significant test

Based on evaluator reports, some image markers were also found to be associated with a particular lesion type (Fig. 5). *Lobules* (OR 143.2, CI 62.9;326.1, *p* < 0.001), *clefting* (OR 158.7, CI 64.5; 390.7, *p* < 0.001) and *collagen alterations* (OR 12, CI 6.1; 23.3, *p* < 0.001) were strongly associated with BCC, as was the non-presence of *severe dysplasia* (OR 0.08, CI 0.04;0.2, *p* < 0.001) and *tumor budding* (OR 0.4, CI 0.3;0.7, *p* < 0.001). Meanwhile, *interrupted DEJ* (OR 2.7, CI 1.6; 4.4, *p* < 0.001), *keratin pearls* (OR 2.5, CI 1.3;4.5, *p* < 0.004), and *broad strands* (OR 1.9, CI 1.2; 3.1, *p* = 0.009), were associated with SCC, in addition to the non-presence of *clefting* (OR 0.3, CI 0.2;0.6, *p* < 0.001), *lobules* (OR 0.3, CI 0.2;0.5, *p* < 0.001), and *well-defined DEJ* (OR 0.4, CI 0.2;0.6, *p* < 0.001).

The presence of *severe dysplasia* (OR 7.1, CI 3.9; 13.2, *p* < 0.001) and surprisingly *interrupted DEJ* (OR 2.5, CI 1.3;4.9, *p* = 0.005) was associated with CIS, as was the non-presence of *clefting* (OR 0.08, CI 0.03;0.2, *p* < 0.001), *lobules* (OR 0.05, CI 0.01;0.2, *p* < 0.001), and *mild-moderate dysplasia* (OR 0.4, CI 0.2;0.8, *p* = 0.005). Finally, *tumor budding* (OR 2.3, CI 1.5;3.5, *p* < 0.001), *well-defined DEJ* (OR 2.1, CI 1.3;3.3, *p* = 0.003), and interestingly *severe dysplasia* (OR 1.9, CI 1.3;2.9, *p* = 0.002) were associated with AK; in contrast to *lobules* (OR 0.09, CI 0.04;0.2, *p* < 0.001), *clefting* (OR 0.1, CI 0.06;0.2, *p* < 0.001), *collagen alterations* (OR 0.4, CI 0.2;0.6, *p* < 0.001) and *interrupted DEJ* (OR 0.5, CI 0.3;0.8, *p* = 0.003) which were inversely associated with AK.

## Discussion

This study examined IOA among six evaluators new to LC-OCT, based on assessment of 10 key LC-OCT image markers in BCC, SCC, CIS, and AK images. The study found varying IOA, ranging from overall κ values of 0.06 to 0.68 depending on image marker (Fig. 2). The highest IOAs were noted for *lobules* and *clefting* (substantial κ) as well as *severe dysplasia* (moderate κ). In contrast, the remaining seven image markers were observed with slight and fair κ values across all evaluators. Notably, for both experienced and less experienced evaluators, assessments of DEJ integrity had consistently low κ values. The tendency among evaluators to agree on the presence or absence of *lobules*, *clefting*, *severe dysplasia*, may suggest that these image markers are easily and consistently recognized by LC-OCT users irrespective of their past imaging experience, whereas markers related to the DEJ appears to be challenging.

The higher IOA observed for *lobules*, compared to other image markers in the study, could reflect its distinct appearance as well as factors that enable a strong image-signal, including often superficial skin location and occasional occurrence with an atrophic epidermis. Described by Suppa et al., *lobules* often exhibit the combination of a central and characteristic *millefeuille* LC-OCT pattern and prominent peripheral dark *clefting* surrounded by a bright rim indicating *collagen alterations*. The combination of these three markers are referred to as *triad of colors* [23]. Furthermore, *lobules* often occur in combination with an atrophic epidermis in nodular BCC [25]. This atrophy reduces the distance from the skin surface to the dermis, potentially making the signal and image definition surrounding *lobules*, stronger. Indeed, previous studies on LC-OCT imaging of skin cancer lesions report a decrease in image definition with increasing depth [23, 38, 39], with cellular markers visible up to 300 μm depth, and architectural markers visible up to 400 μm depth [38]. This could similarly explain why *severe dysplasia*, with its distinct appearance in the uppermost layer of the epidermis where image signal/cellular details often are most clear (see Fig. 1A), had the third highest κ; despite reported challenges associated with examining single cell atypia using LC-OCT [40].

Lower κ values could be due to certain image markers being inherently difficult to identify or challenges distinguishing between two markers. For example, the thin and subtle line of a *well-defined DEJ* (Fig. 1D) is not easy to detect, particularly in the presence of hyperkeratosis and acanthosis seen in KC lesions [19, 21, 22, 41]. A discrete break in the DEJ caused by invasive tumor strands, and their presence as *broad strands* in the dermis, is arguably also challenging to visualize (Fig. 1E **and** Fig. 1F). *Tumor budding* can resemble *lobules* connected to the epidermis, conceivably making it difficult for evaluators to distinguish the two markers (i.e., both image markers are visible as a protrusion of the epidermis (Fig. 1H, top image, vs Fig. 1C). Another reason for lower κ values may be related to differing understandings of image marker definitions, especially since the validity of κ values depend upon an detailed definition of concordant or discordant findings [42]. This study used predefined image markers described in a published review of LC-OCT literature [28], but expert consensus in the LC-OCT community on image marker definitions is lacking. Comparative studies between LC-OCT and histopathology, as is reported for atypical keratinocyte growth patterns in AK [20], may establish a shared understanding of what should, for example, be deemed an *interrupted DEJ* or a *broad strand* in LC-OCT images. Nevertheless, it appears that background experience in OCT/RCM imaging can somewhat increase κ values, observer’ confidence, and resistance to image-artefacts for most image markers.

Some κ values were affected by κ paradox [32, 33]. Although we aimed to include images with a 50% prevalence rate of image markers, a large imbalance between evaluator-reported prevalence was observed for *mild-moderate dysplasia*, *broad-strands*, and *keratin pearls* (Table S2). In addition, some markers displayed notable differences between their P_pos_ and P_neg_, meaning evaluators had good agreement on presence but not absence, or vice versa. Therefore, the κ values of *mild-moderate dysplasia*, *broad-strands*, and *keratin pearls* should be interpreted with caution as the estimated κ may be lowered.

Importantly, this study did not assess the diagnostic utility of LC-OCT, but the frequency of image markers for each lesion-type reported by evaluators new to the technology. Therefore, the true prevalence of the image markers within KC and precursor lesions remains to be tested by experts in the field. Nevertheless, in line with previous reports, we found *lobules*,* clefting*, and *collagen alterations* significantly associated with BCC [23–25]; *interrupted DEJ*,* broad strands* and *keratin pearls* with SCC, and *severe dysplasia* with CIS [21, 22, 43], as well as *well-defined DEJ* and *tumor budding* with AK [20]. Unexpected associations, such as an *interrupted DEJ* in CIS, could be attributed to the smaller sample size of CIS images (*n* = 12 image cases) as compared to other lesion-types (*n* = 21 for AK, SCC, and BCC) or the aforementioned challenges related DEJ assessment. Another unexpected finding was the association between *severe dysplasia* and AK: we speculate that this could be due to the presence of single, large cells in the stratum granulosum, a feature that is infrequently observed in AK lesions that would not tip the histopathology diagnosis towards CIS, a lesion characterized by more extensive full-thickness epidermal dysplasia in traditional histopathology. While the reference standard was histopathology, it is also possible that an area of CIS was seen in the LC-OCT image but not captured in the biopsy specimen.

This study has some limitations. We used the poor-almost perfect scale for κ interpretation [31], although other approaches are available [44]. Our study was further based on a limited selection of KC and precursors images, which limits the generalizability of our results. Future studies should include an extensive database to ensure sufficient prevalence of rare image markers. Additionally, we did not take the impact of in-person versus virtual image-evaluation into account, although this aspect may be important to image quality. Study strengths, on the other hand, are related to the study’s real-world applicability. We assessed six evaluators from three different centers with varying degrees of imaging experience, a selection of evaluators that in many ways is representative of the current, fledgling LC-OCT field.

## Conclusion and perspectives

In conclusion, our study highlights variations in IOA between evaluators without prior experience with LC-OCT in assessment of 10 key LC-OCT image markers in SCC, BCC, CIS, and AK lesions. For *lobules* and *clefting*, as well as, *severe dysplasia*, evaluators achieved higher IOA, while lowest IOA was shown for assessments of DEJ integrity. The study thus provides new insights into imaging-based lesion assessment in dermato-oncology and specifically, shows the degree of IOA in LC-OCT image marker assessment amongst new users of this increasingly implemented technology.

## Electronic supplementary material

Below is the link to the electronic supplementary material.

Supplementary material 1

## Data Availability

No datasets were generated or analysed during the current study.
