# Supplementary material for: Interobserver agreement on line-field confocal optical coherence tomography image markers in keratinocyte carcinomas and precursor lesions
Source: Arch Dermatol Res. 2024 Sep 6;316(8):608. doi: 10.1007/s00403-024-03344-y (PMC11379787; doi:10.1007/s00403-024-03344-y)
Supplement: Supplementary file 1 — Supplementary material 1 [file 403_2024_3344_MOESM1_ESM.docx]

**Supplementary Material**

**
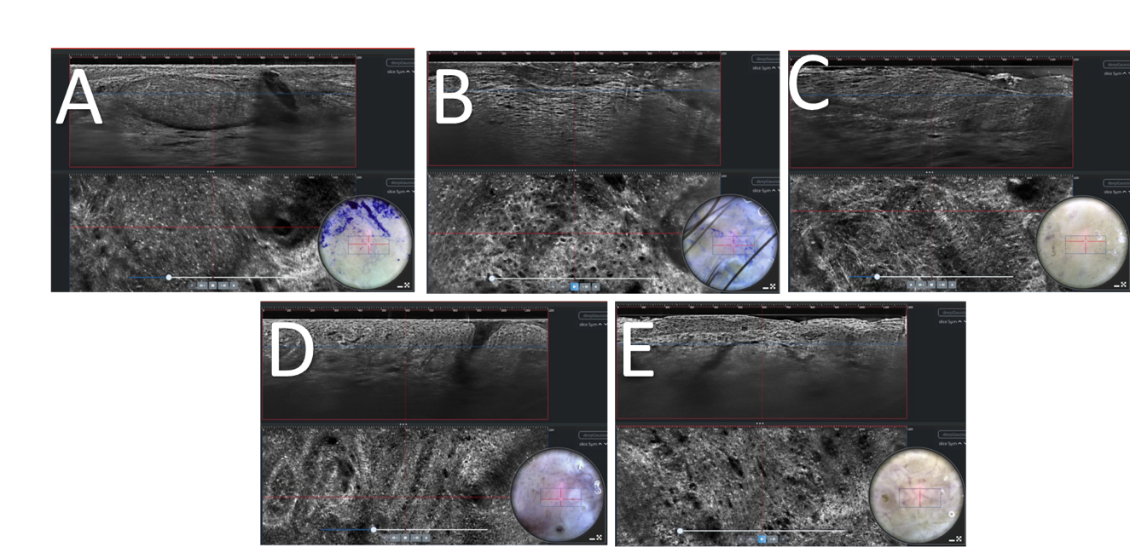
**

**Figure S1: Line-field confocal optical coherence tomography images (cross-section and en-face) of keratinocyte carcinomas, precursor lesions, and healthy skin.**
A) Basal cell carcinoma, B) squamous cell carcinoma, C) squamous cell carcinoma in situ, D) actinic keratosis, and E) healthy skin.

| **Experienced or novice device user in optical imaging** | **Evaluator** | **Did the evaluator complete a skin cancer optical coherence tomography e-learning module** | **Has the evaluator published any studies on non-invasive skin cancer imaging?** |
| --- | --- | --- | --- |
| Experienced | Evaluator 1 | Yes | Yes |
|  | Evaluator 2 | Yes | Yes, |
|  | Evaluator 3 | Yes | Yes |
| Novice | Evaluator 4 | No | Yes |
|  | Evaluator 5 | No | Yes |
|  | Evaluator 6 | No | Yes |

**Table S1. Evaluators with experience in conventional optical coherence tomography and/or reflectance confocal microscopy skin cancer imaging.**
Categorized as experienced or novice device users in optical imaging based on whether or not evaluators completed a skin cancer optical coherence tomography internal e-learning module.

| **IOA for all evaluators (n=6)** | **Conger Kappa (95% CI)** | | **Proportion of observed agreement (Po)** | | **Proportion of specific positive agreement (Ppos)** | **Proportion of specific negative agreement (Pneg)** | **Ppos-Pneg** | **f_1_-f_2_** |
| --- | --- | --- | --- | --- | --- | --- | --- | --- |
| Severe dysplasia | 0.42 (0.31; 0.53) | | 0.72 (0.66;0.77) | | 0.67 | 0.75 | -0.09 | -11 |
| **Mild-moderate dysplasia** | **0.11 (0.02;0.2)** | | **0.60 (0.54;0.64)** | | **0.41** | **0.70** | **-0.30** | **-24** |
| Tumor budding | 0.21 (0.11;0.30) | | 0.59 (0.52;0.62) | | 0.56 | 0.62 | -0.05 | -5 |
| Well-defined DEJ | 0.07 (0;0.15) | | 0.57 (0.54;0.69) | | 0.59 | 0.54 | 0.05 | 4 |
| Interrupted DEJ | 0.06 (-0.002;0.13) | | 0.57(0.44;0.69) | | 0.52 | 0.60 | -0.08 | -5 |
| **Broad strands** | **0.13 (0.05;0.21)** | | **0.70 (0.65;0.75)** | | **0.31** | **0.81** | **-0.51** | **-43** |
| **Keratin Pearls** | **0.24 (0.04;0.43)** | | **0.85 (0.81;0.90)** | | **0.32** | **0.92** | **-0.60** | **-59** |
| Lobules | 0.68 (0.57;0.78) | | 0.86 (0.81;0.91) | | 0.78 | 0.90 | -0.11 | -26 |
| Clefting | 0.63 (0.52;0.74) | | 0.83 (0.78;0.88) | | 0.77 | 0.87 | -0.10 | -21 |
| Collagen alterations | 0.32 (0.22;0.42) | | 0.68 (0.62;0.73) | | 0.73 | 0.59 | 0.15 | 16 |
| **IOA for experienced (n=3)** |  |  | |  |  |  |  |  |
| Severe dysplasia | 0.49 (0.35; 0.64) | | 0.75 (0.68;0.82) | | 0.70 | 0.79 | -0.09 | -14 |
| **Mild-moderate dysplasia** | **0.25 (0.08;0.41)** | | **0.72 (0.65;0.80)** | | **0.45** | **0.82** | **-0.37** | **-38** |
| Tumor budding | 0.25 (0.10;0.40) | | 0.61 (0.50;0.65) | | 0.60 | 0.62 | -0.02 | -2 |
| Well-defined DEJ | 0.27 (0.11;0.43) | | 0.66(0.54;0.78) | | 0.72 | 0.56 | 0.16 | 14 |
| Interrupted DEJ | 0.24 (0.07;0.41) | | 0.67(0.54;0.79) | | 0.53 | 0.74 | -0.21 | -16 |
| **Broad strands** | **0.12 (-0.05;0.29)** | | **0.72 (0.64;0.79)** | | **0.27** | **0.82** | **-0.55** | **-46** |
| **Keratin Pearls** | **0.24 (-0.02;0.50)** | | **0.85 (0.79;0.91)** | | **0.32** | **0.92** | **-0.60** | **-58** |
| Lobules | 0.77 (0.64;0.90) | | 0.90 (0.85;0.96) | | 0.83 | 0.93 | -0.10 | -31 |
| Clefting | 0.74 (0.61;0.87) | | 0.88 (0.82;0.94) | | 0.82 | 0.91 | -0.09 | -25 |
| Collagen alterations | 0.36 (0.22;0.50) | | 0.68 (0.61;0.75) | | 0.73 | 0.62 | 0.11 | 12 |
|  |  | |  | |  |  |  |  |
| **IOA for novices (n=3)** |  |  | |  |  |  |  |  |
| Severe dysplasia | 0.41 (0.26; 0.55) | | 0.71 (0.63;0.78) | | 0.67 | 0.74 | -0.07 | -8 |
| Mild-moderate dysplasia | 0.16 (0.01;0.30) | | 0.58 (0.48;0.63) | | 0.50 | 0.64 | -0.14 | -11 |
| Tumor budding | 0.15 (0.01;0.28) | | 0.57 (0.49;0.63) | | 0.52 | 0.61 | -0.09 | -8 |
| Well-defined DEJ | - 0.11 (-0.23;0.004) | | 0.46(0.33;0.59) | | 0.39 | 0.52 | -0.13 | -7 |
| Interrupted DEJ | - 0.10 (-0.23;0.02) | | 0.47(0.34;0.59) | | 0.52 | 0.40 | 0.13 | 7 |
| **Broad strands** | **0.06 (-0.06;0.19)** | | **0.66 (0.58;0.73)** | | **0.27** | **0.78** | **-0.51** | **-40** |
| **Keratin Pearls** | **0.18 (-0.04;0.41)** | | **0.85 (0.79;0.91)** | | **0.26** | **0.92** | **-0.65** | **-60** |
| Lobules | 0.63 (0.50;0.77) | | 0.83 (0.77;0.90) | | 0.73 | 0.87 | -0.11 | -22 |
| Clefting | 0.57 (0.43;0.71) | | 0.80 (0.73;0.87) | | 0.73 | 0.84 | -0.10 | -18 |
| Collagen alterations | 0.24 (0.1;0.37) | | 0.64 (0.62;0.73) | | 0.72 | 0.51 | 0.21 | 20 |

**Table S2: Interobserver agreement (IOA) and kappa paradoxes**

In this study, IOA is expressed through Conger kappa (κ) with 95% confidence intervals (31). IOA was tested among six evaluators with optical coherence tomography and/or reflectance confocal microscopy experience (three experienced and three novices), assessing the presence or absence of 10 predefined key line-field confocal optical coherence tomography image markers. These key image markers corresponded to diagnostic important histopathology markers for basal cell carcinoma, squamous cell carcinoma, squamous cell carcinoma in situ/carcinoma in situ, and actinic keratosis and were identified in a previous study (29).
To reveal κ paradoxes, where high agreement but low κ values are found (33), the proportion of observed agreement (P_o_) with 95% CI, the specific positive agreement (P_pos_), the specific negative agreement (P_neg_), as well as the marginal totals f_1_ and f_2_ were calculated. The net product of the absolute difference between P_pos_ and P_neg_ (i.e., P_pos -_ P_neg_), as well as between f_1_ and f_2_ (i.e., f_1_- f_2_), constitutes the main source of the κ paradox (33,34).

In the table, prominent κ paradoxes are identified for *mild-moderate dysplasia, keratin pearls*, and *broad strands*, where the net effect of large inequalities between P_pos_ and P_pneg_, as well as between f_1_ and f_2_, resulted in high Po but a dramatic lowering of the κ value (image markers highlighted in bold).
P_o,_ P_pos,_ P_neg_, f_1_, and f_2_  was derived from a 2x2 table $\frac{a}{c}\frac{b}{d}$ for two evaluators and extended to include all combinations of evaluators using provided formulas (37,38). Here, *a* denotes the agreement on the presence of an image marker, *d* the agreement on the absence of an image maker, and *b* and *c* the disagreement on the presence or absence of an image marker. P_o_ represent the overall raw agreement for both the presence and absence of an image marker (formula for two evaluators, (*a* + *d)*/(*a* + *b* + *c* + *d*). Unlike κ, P_o_ is not a chance-corrected agreement measure. P_pos_ and P_neg,_ represents the two components of P_o_, they show the agreement on either the presence or absence of an image marker, respectively (formula for two evaluators, P_pos_ = (*2a)/(2a+b+c) and*  P_neg_ = (*2d)/(2d+b+c)).* f_1_ and f_2_ represent evaluator-reported prevalence on marker presence or absence, respectively (formula for two evaluators, f_1_ = *a+c,*  f_2_= *b+d*). Only the absolute difference between f_1_ and f_2_ (f_1_-f_2_) are reported in the table.
